# Quantitative Analysis of Bronchiectasis in Alpha‐1 Antitrypsin Deficiency

**DOI:** 10.1155/carj/5419612

**Published:** 2026-06-24

**Authors:** Joshua De Soyza, Philipp Höger, Paul Ellis, Oliver Weinheimer, Franziska Trudzinski, Alice M. Turner

**Affiliations:** ^1^ Department of Applied Health Sciences, University of Birmingham, Birmingham, UK, birmingham.ac.uk; ^2^ Department of Respiratory Medicine, University Hospitals Birmingham, Birmingham, UK, nhs.uk; ^3^ Department of Pneumology and Critical Care Medicine, Thoraxklinik, Heidelberg, Germany; ^4^ Translational Lung Research Center Heidelberg (TLRC), University of Heidelberg, Heidelberg, Germany, uni-heidelberg.de; ^5^ Department of Diagnostic and Interventional Radiology, University Hospital of Heidelberg, Heidelberg, Germany, heidelberg-university-hospital.com

## Abstract

**Introduction:**

Alpha‐1 antitrypsin deficiency (AATD) may be directly related to bronchiectasis. However, radiological diagnosis of mild bronchiectasis is subjective, and visual assessment of airway tapering is time‐consuming. CT algorithms have been used to quantify airway tapering in other bronchiectasis aetiologies. We assessed whether this process can be used in AATD.

**Methods:**

Historic CT scans from patients with severe AATD in the Birmingham registry were processed with YACTA software (Weinheimer et al., 2017), and data on bronchiectasis index (BEI), airway wall thickness (AWPi10) and emphysema (PD15) were extracted. Correlation with visual data was assessed using standard statistical tests. Receiver operating characteristic (ROC) curves were used to find thresholds at which algorithm data are associated with visual data. Regression models assessed the impact of algorithm data on lung function decline, exacerbation rate, mortality and bronchiectasis severity index (BSI), corrected for age, sex, smoking status and PD15.

**Results:**

A total of 154 scans were analysed. BEI correlated moderately well with visual scoring of bronchial dilatation (*p* < 0.001), bronchial wall thickness (BWT, *p* < 0.001), number of lobes affected (*p* < 0.001) and morphology (*p* < 0.001). AWPi10 did not correlate with BWT (*p* = 0.28). Thresholds were identified for the association of BEI with bronchial dilatation ≥ 2 × arterial diameter; lobar involvement ≥ 3 lobes, and presence of varicose or cystic morphology, though sensitivity and specificity were relatively low. BEI is associated only with BSI (*p* = 0.002). AWPi10 did not associate with clinical outcomes.

**Conclusion:**

Algorithm CT analysis has value in quantifying mild bronchiectasis in AATD and may be used in trials of emerging therapies.

## 1. Introduction

Alpha‐1 antitrypsin deficiency (AATD) is a rare disease which causes emphysema by unopposed activity of neutrophil elastase, among other proteases [1, 2]. Exhibiting autosomal co‐dominant inheritance, the most common severely deficient genotype is PiZZ, with the PiSZ genotype causing less severe disease and thought to cause lung disease only with concurrent smoking [3, 4].

Increased neutrophil elastase activity is also heavily implicated in the pathogenesis of bronchiectasis of all causes [5, 6], and bronchiectasis is seen in a significant minority of patients with AATD, even without concurrent COPD [7]. Conversely, AATD has recently been identified in 2.8% of patients with bronchiectasis without emphysema [8]. A direct association between AATD and bronchiectasis has been suggested, including in data previously published by our group, which saw an inverse correlation between serum AAT and bronchiectasis diagnosis, which was independent of concurrent COPD diagnosis [9, 10].

However, most cases of AATD‐bronchiectasis are radiologically mild, affecting only a minority of lobes, with predominantly cylindrical Reid morphology, and with only minor dilatation relative to the accompanying artery [9, 10]. Furthermore, in all‐cause bronchiectasis, the radiological appearance of bronchiectasis has only been shown to affect clinical outcomes when there is involvement of > 3 lobes, or cystic morphology [11, 12].

This raises the issue of the diagnosis and classification of mild bronchiectasis, which has been shown to be subjective [13]. All current scoring systems rely on visual assessment of bronchial dilatation (BD) relative to the diameter of the accompanying bronchial artery, but in the case of pulmonary hypertension, the pulmonary arteries may themselves be enlarged, meaning that a dilated bronchiole may not appear so in relation to the hypertrophied and dilated adjacent pulmonary artery. Pulmonary hypertension has many causes, including bronchiectasis and COPD, and occurs in a minority of all such patients [14–16], raising the possibility of underdiagnosis of bronchiectasis in these patients. Bronchial arterial diameter can also vary widely even in health [17]. The identification of lack of airway tapering may be superior to comparison with adjacent arteries [18] but is time‐consuming and requires expert radiological skill [19]. The use of automated quantitative airway analysis may minimise the time and requirement for expertise by analysing vast quantities of data with objectivity. To date, some software packages have been tested and validated in other respiratory disorders but lack validation in bronchiectasis [20, 21]. One example is the Yet Another CT Analyser (YACTA) software [4], which is rare among such software tools in being able to construct a true tapering index for the whole lung. Parameters calculated during YACTA analysis have been found to associate with FEV_1_% predicted in adults with cystic fibrosis [21]: in particular, the airway parameters showed a high sensitivity to detect the smallest changes. Airway and parenchymal parameters from YACTA have also been associated with clinical outcomes in COPD, including GOLD severity [22], lung function decline [23], mortality [24] and to differ between smokers and nonsmokers [25]. We therefore aimed to assess whether the use of YACTA might make it possible to objectively assess bronchiectasis in AATD and, therefore, improve on the wide‐ranging estimates of its prevalence and association with AATD.

## 2. Methods

### 2.1. Patient Data

Patients who have AATD and are registered to the Birmingham AATD registry were included, as described previously [9]. The study was ethically approved (approval reference numbers 3359a and 18/SC/0541), and all patients gave informed consent. In brief, patients underwent annual clinical assessment including lung function and assessment of exacerbation rate. CT scanning of the lungs was performed in the earlier years of the cohort as part of research and in later years only when clinically indicated. This analysis pertains only to CT scans of the cohort. All CT scans were taken in a stable state, i.e., not during exacerbation or acute infection.

### 2.2. Imaging

**FIGURE 1:**
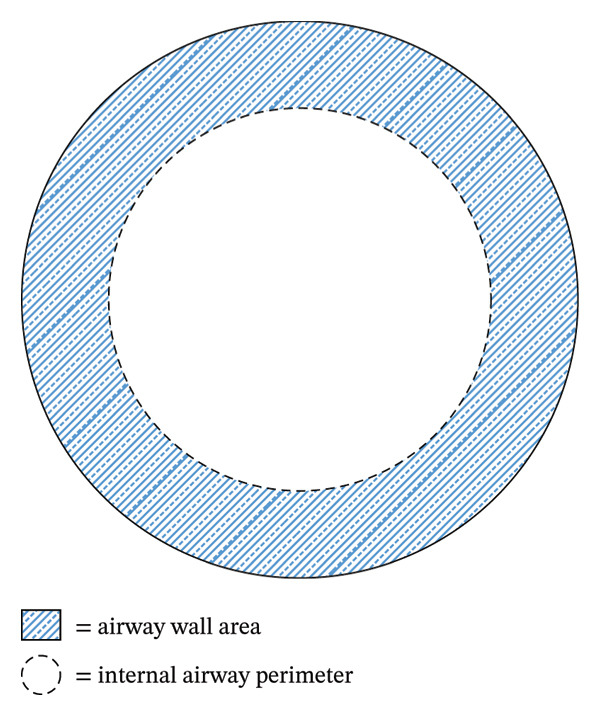
Schematic demonstrating derivation of airway wall area.

CT scans which had been systematically analysed visually in our previous published work [9] were assessed with the YACTA software (Version 2.9.4.114), as described in Weinheimer et al. [4]. Only scans with a slice thickness of ≤ 1.5 mm were examined; in practice this excluded older scans, as imaging protocols changed over time, in common with the wider healthcare system. Of the wide variety of quantitative CT (QCT) parameters assessed by YACTA, three were selected. Firstly, bronchiectasis index (BEI), which is a measure of airway tapering assessed by measuring the transverse diameter of the airway in the plane of that airway [4]. Secondly, AWPi10 (Figure 1), a standardised airway wall thickness measure [24, 26]. This is done by segmenting the entire tracheobronchial tree and determining the airway geometry of all segmented bronchi by a parameter‐free integral‐based method (IBMpf) [4, 27]. The square root of airway wall areas is plotted against the inner perimeter of the airway for each airway location. A regression line is determined through the point cloud, and this is used to derive the wall thickness for a hypothetical airway with a 10 mm internal perimeter. This variable was selected, as it has been used in recent studies as a standardised measure of airway wall thickness, with a weak to moderate correlation with bronchiectasis severity index (BSI) in one study [28–30].

Thirdly, PD15 was selected, which is a quantitative measure of density, measured in Hounsfield units, representing the value below which 15% of the lung has lower density. It has been established as a reliable measure of lung density in both usual and AATD–COPD since 2004 [31–33].

### 2.3. Statistical Analysis

Analysis aimed to assess, firstly, whether the YACTA parameters correlated with visual assessment of the CT scans as previously published [9]; secondly, whether thresholds of the continuous YACTA data could be identified to diagnose bronchiectasis with an acceptable level of sensitivity and specificity; and, thirdly, whether the YACTA parameters correlated with the clinical outcomes of interest. Inferential statistics relating YACTA parameters to clinical outcomes were limited to those with severe AATD, i.e., those with PiZZ or genotypes producing similarly low levels of AAT. All statistical analysis was done using R Version 4.3.2 in RStudio 2024.04.2.

### 2.4. Correlation With 5 Binary Ion Between YACTA Parameters and Visual CT Assessment

For the assessment of correlation between YACTA and visual CT scan assessment, standard statistical tests were used. Student’s t‐test or one‐way analysis of variance for comparison of means between groups, and Wilcoxon rank sum or Kruskal–Wallis tests for comparison of binary groups where data was not normally distributed. The BEI was compared with visual assessment of number of lobes affected, morphology, BD and bronchial wall thickening. Morphology type was converted to an ordinal variable with value 0 if no bronchiectasis is present; 1 if only cylindrical morphology is present; 2 if varicose but not cystic morphology is present, and 3 if cystic morphology is present. The AWPi10 YACTA parameter was compared with visual assessment of bronchial wall thickening only.

### 2.5. Identification of YACTA Thresholds Correlating With Visual Assessment

For the assessment of thresholds which correlated with bronchiectasis, receiver operating characteristic (ROC) curves were calculated. If the 95% confidence interval of the area under the curve (AUC) included 50%, no further analysis was conducted; if the AUC 95% confidence interval range did not include 50%, the optimal threshold was identified using Youden’s J method. This analysis was performed firstly for the ability of BEI to identify the following visual assessment parameters: bronchiectasis of any kind; cystic morphology; varicose or cystic morphology; bronchial wall thickening at each grade; BD at each grade and number of lobes affected. Secondly, ROC analysis was conducted for the association of the BEI with bronchiectasis in each respective lobe. Thirdly, ROC analysis was also conducted for the association of AWPi10 with bronchial wall thickening scores of each grade.

### 2.6. Correlation of YACTA Parameters With Clinical Outcomes

For the assessment of correlation between YACTA parameters and clinical outcomes, regression models were calculated. These used linear regression for FEV_1_, KCO and TLCO percent predicted yearly decline, FEV_1_ baseline percent predicted and FEV_1_ mL yearly decline; zero‐inflated logistic regression models were used for exacerbation rate analysis. These models were only conducted on patients with severe AATD (PiZZ or rare genotypes producing similarly low serum AAT levels).

## 3. Results

### 3.1. Algorithmic Analysis of CT Bronchiectasis

#### 3.1.1. Summary Statistics

A total of 154 of 290 available scans met inclusion criteria of slice thickness. Summary statistics are provided in Table 1. Visual and algorithmic radiology data are summarised in Table 2.

**TABLE 1:** Summary statistics for patients with CT scans assessed by YACTA.

	Overall
*n*	154
Age (years)	52.46 (10.57)
Sex (male, %)	73 (47.4)
BMI (kg/m^2^)	26.17 (5.28)
Genotype (%)	
ZZ	138 (89.6)
SZ	11 (7.1)
Other severe (MMalton‐Null, Z‐null and FZ)	3 (1.8)
Other nonsevere (SS, MZ)	2 (1.2)
Ever smoker	107 (69.9)
COPD	117 (76.0)
Annualised exacerbation rate	1.04 (1.39)
Baseline FEV_1_% predicted	60.65 (27.74)
Baseline FVC% predicted	94.83 (22.55)
Baseline FEV_1_/FVC	0.51 (0.20)
Baseline KCO% predicted	74.37 (23.54)
Annualised FEV_1_% predicted change	−0.73 (2.16)
Annualised KCO% predicted change	−1.70 (2.02)

**TABLE 2:** Summary of visual and algorithmic radiology data for patients with scans assessed by YACTA.

Visual Bronchial Dilatation Score (%)	
0	55 (35.7)
1	75 (48.7)
2	17 (11.0)
3	8 (5.2)
Visual Bronchial Wall Thickness Score (%)	
0	55 (35.7)
1	76 (49.4)
2	18 (11.7)
3	5 (3.2)
Number of Lobes Affected (%)	
0	55 (35.7)
1	15 (9.7)
2	57 (37.0)
3	6 (3.9)
4	6 (3.9)
5	1 (0.6)
6	14 (9.1)
Visual Morphology Score (%)	
0: None	54 (35.1)
1: Cylindrical only	78 (50.6)
2: Varicose ( ± cylindrical)	19 (12.3)
3: Cystic ( ± varicose or cylindrical)	3 (1.9)
Bronchiectasis index (BEI) (median [IQR])	0.63 (0.78)
AWPi10 (mean [SD])	0.19 (0.08)
PD15 (mean [SD])	−946.5 (37.03)

**FIGURE 2:**
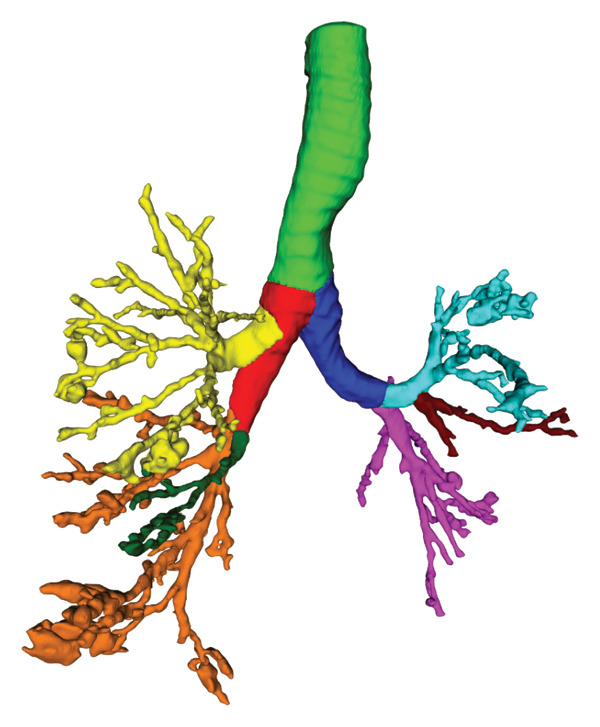
Algorithm‐generated 3‐dimensional airway model exhibiting bronchiectasis in all 6 lobes.

For patients with outlying BEI values, 3‐dimensional models generated by the algorithm were visually inspected to ensure the algorithm had appropriately identified the airway. This element of quality control was recommended by the original authors of the software. An example of such a 3‐dimensional model is given in Figure 2.

#### 3.1.2. Comparison of Algorithmic With Visual Data

BEI of the whole lung was significantly higher in those with undifferentiated bronchiectasis identified on visual assessment vs those without (median BEI 0.88 vs 0.50, Wilcoxon rank sum estimate −0.26, 95% CI −0.46 to −0.09, *p* = 0.002).

#### 3.1.3. Comparison With Visual BD Score

**FIGURE 3:**
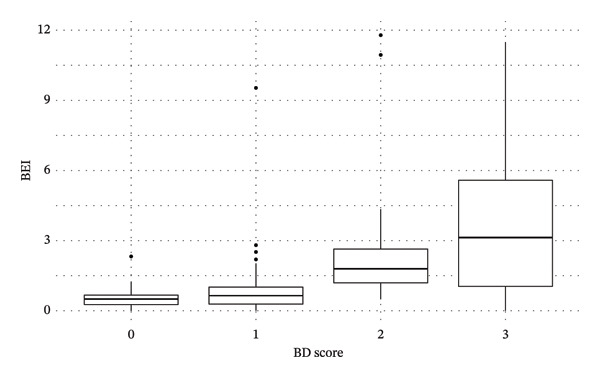
Box plot of bronchiectasis index (BEI) by visual bronchial dilatation (BD) score.

BEI was correlated with visual BD score (Figure 3, *χ* ^2^ = 38.47, *p* < 0.001).

#### 3.1.4. Comparison With Visual BWT Score

BEI is also correlated with visual BWT score (*χ* ^2^ = 20.1, *p* < 0.001). AWPi10 did not correlate with BWT (*χ* ^2^ = 3.88, *p* = 0.28).

#### 3.1.5. Comparison With Morphology

**FIGURE 4:**
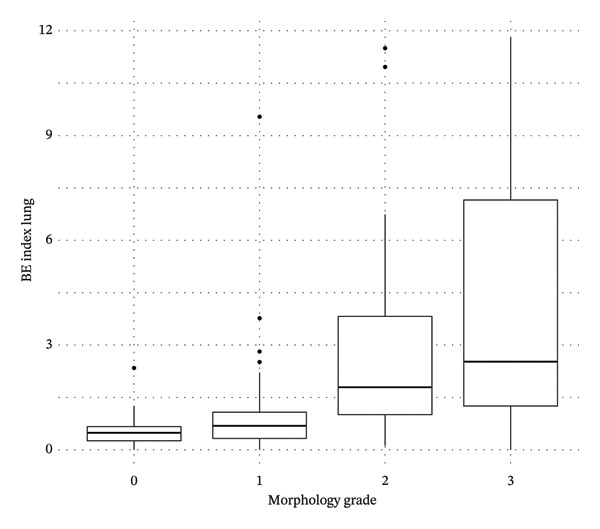
Box plot of BEI by bronchiectasis morphology.

BEI correlated with morphology grade, with *χ* ^2^ = 21.883 (Figure 4, *p* < 0.001).

#### 3.1.6. Comparison With Lobar Distribution

**FIGURE 5:**
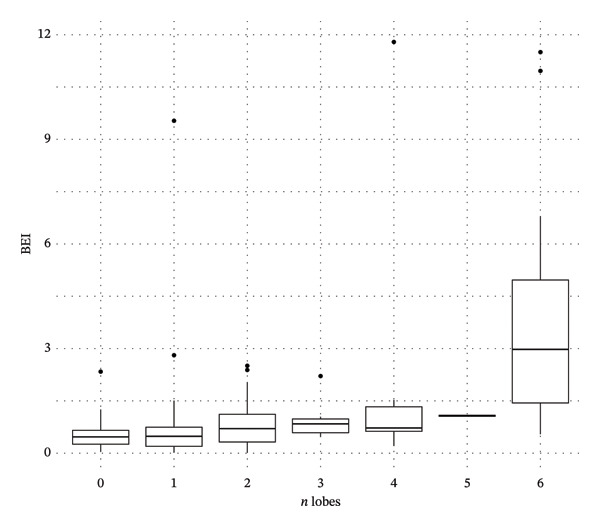
Box plot of BEI by number of lobes affected with bronchiectasis on visual assessment.

BEI is also associated with the number of lobes affected (Figure 5, *χ* ^2^ = 0.37, *p* = <0.001). Univariate linear regression analysis revealed an increase of 0.51 in BEI for each extra lobe affected (95% CI 0.36 –0.66, *R* ^2^/*R* ^2^ adjusted 0.236/0.231, *p* < 0.001).

BEI was also analysed in relation to the cumulative number of affected lobes, simplified into binary metrics indicating the involvement of at least ‘*n’* lobes. For instance, BEI was compared to whether at least 2, 3, 4, 5 or all 6 lobes were affected. Results of this analysis are shown in Table 3. The effect size increased with each additional lobe involvement, with a high degree of statistical significance et al. levels.

**TABLE 3:** BEI association with cumulative lobar involvement on visual assessment by the Wilcoxon rank sum test.

BEI			
Lobar involvement	Estimate	95% CI	*p*
≥ 2	0.38	0.20–0.58	**<** **0.001**
≥ 3	0.82	0.48–1.57	**<** **0.001**
≥ 4	1.25	0.64–2.49	**<** **0.001**
≥ 5	2.14	1.02–3.27	**<** **0.001**
6	2.33	1.24–3.54	**<** **0.001**

*Note:* Bold values are used to highlight statistically significant results.

AWPi10 did not correlate with visual BWT score (χ^2^ = 3.88, *p* = 0.28).

Thresholds for the association of algorithm‐derived continuous data with visual assessment of bronchiectasis.

ROC analysis was performed for YACTA variables, where these variables showed correlation with visual parameters in the analyses above. ROC was also performed for BEI as a measure of visual bronchiectasis of any kind.

Despite not showing an association on linear regression, ROC analysis of BEI and visual diagnosis of bronchiectasis showed an AUC of greater than 50% (64.3%, 95% CI 55.6%–73%), demonstrating some ability of BEI to discriminate between the classes. The optimum threshold of BEI for the diagnosis of bronchiectasis by Youden’s J method was 0.76, with 81.03% sensitivity but only 50% specificity (Youden’s J statistic [*J*] = 0.31).

### 3.2. ROC Analysis of Visual BD Score

**FIGURE 6:**
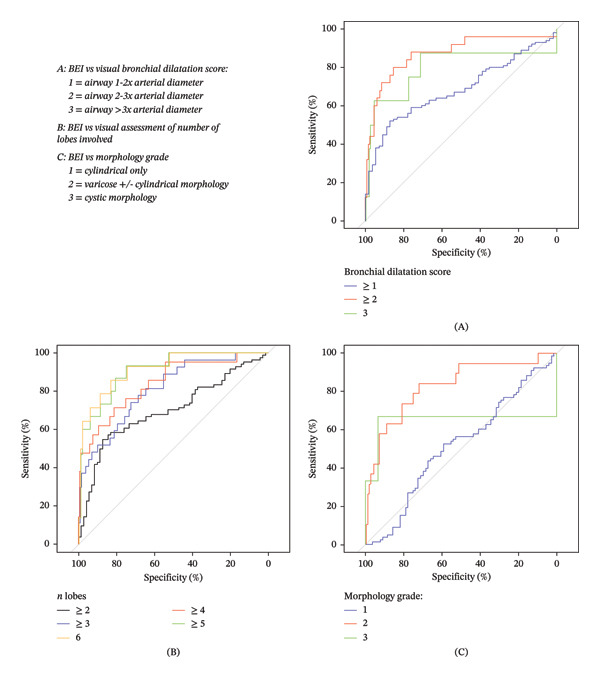
(A–C) ROC analyses for the association of BEI with visual CT scoring. (A) BEI vs visual bronchial dilatation score: 1 = airway 1‐2 × arterial diameter. 2 = airway 2‐3 × arterial diameter. 3 = airway > 3× arterial diameter. (B) BEI vs visual assessment of number of lobes involved. (C) BEI vs morphology grade. 1 = cylindrical only. 2 = varicose ± cylindrical morphology. 3 = cystic morphology.

The visual BD score was compared with the BEI, with the strongest result for the BD ≥ 2 level (AUC 81.2%, 95% CI 68.9% – 93.5%, Figure 6A). The optimal threshold for BEI association with BD ≥ 1 was 0.76 (sensitivity 52%, specificity 87.04%, *J* 0.39), BD ≥ 2 optimal threshold was 1.07 (sensitivity 80%, specificity 85.3%, *J* 0.65), and for BD 3 the optimal threshold was 0.92 (sensitivity 87.5%, specificity 71.2%, *J* 0.58).

### 3.3. ROC Analysis of Lobar Involvement

For lobar involvement, ROC analysis was performed on the association of BEI visual measurements: specifically whether at least 2, 3, 4, 5 or all 6 lobes were affected with bronchiectasis. ROC curves for these 5 visual parameters are shown in Figure 6B, with AUC values in Table 4. Optimal thresholds for each parameter are shown in Table 5. An acceptable level of sensitivity and specificity was achieved for all except the “≥ 2 lobes” and “≥ 3 lobes” parameters. A drop in optimal threshold was observed in the “≥ 5 lobes” category, which is probably anomalous and related to the low number of patients with only 5 lobes affected (Table 5).

**TABLE 4:** ROC AUC values for BEI association with visual assessment of bronchiectasis lobar involvement.

*n* lobes with bronchiectasis	AUC (%)	95% CI (%)
≥ 2	69.8	61.5–78.2
≥ 3	80.9	71.0–89.9
≥ 4	84.0	74.1–93.9
≥ 5	90.6	83.4–97.8
6	91.3	83.8–98.8

**TABLE 5:** Optimal thresholds for the association of BEI with lobar involvement, expressed as 5 different binary parameters.

Number of lobes with visually assessed bronchiectasis	Optimal BEI threshold	Sensitivity	Specificity	Youden’s J statistic
≥ 2	0.81	54.8	87.1	0.42
≥ 3	0.81	74.1	72.4	0.47
≥ 4	1.07	71.4	81.2	0.53
≥ 5	0.92	93.3	74.8	0.68
6	1.10	85.71	82.86	0.69

*Note:* The identical thresholds for both ≥ 2 and ≥ 3 lobes are correct.

### 3.4. ROC Analysis of Morphology

ROC analysis was also conducted on the ability of BEI to identify varicose or cystic morphology. These ROC curves are shown in Figure 6C. Two optimal thresholds were identified for BEI associating with varicose bronchiectasis: BEI 0.84 had 81.8% sensitivity and 72.7% specificity, and BEI 1.07 had 72.7% sensitivity and 81.8% specificity (*J* 54.5 for both). Youden’s *J* was not calculated for cystic bronchiectasis, as ROC curve analysis was not significant (AUC 64.5%, 95% CI 1.2–100).

### 3.5. Comparison of Algorithmic Data With Clinical Outcomes

#### 3.5.1. Comparison With Lung Function

BEI associated with baseline FEV_1_pp on multivariate analysis, with FEV_1_pp being 2.78% higher (*p* = 0.003) for every one‐unit change in BEI (Supporting Table 1). AWPi10 is also associated with baseline FEV_1_pp, with each 0.1 increase in AWPi10 being associated with a 5.1% reduction in baseline FEV_1_pp (95% CI −9.57–−0.64, *p* = 0.025). Smoking status negatively correlated with baseline FEV_1_pp, but there was no association with emphysema as measured by PD15.

However, BEI was not associated with FEV_1_pp decline (estimate −0.03, 95% CI −0.22–0.16, *p* = 0.771), KCOpp (estimate −0.10, 95% −0.29–0.09, *p* = 0.290) or TLCOpp (est. 0.04, 95% CI −0.17–0.24, *p* = 0.708). AWPi10 also did not associate with FEV_1_pp yearly decline (est. −4.811, 95% CI 11.15–1.5, *p* = 0.135), KCOpp yearly decline (est −6.544, 95% CI −14.08–0.99, *p* = 0.88) or TLCOpp yearly decline (est. −3.543, 95% CI −11.65–4.56, *p* = 0.386). Neither BEI nor AWPi10 is associated with exacerbator status or exacerbation rate.

The mean SGRQ score was not associated with BEI (estimate 1.36, 95% CI −2.31–5.02, *p* = 0.463) or AWPi10 (est. 9.43, 95% CI −48.67 to 67.52, *p* = 0.748).

#### 3.5.2. Comparison With BSI

BEI correlated with BSI on multivariate Poisson regression analysis (Supporting Table 2), with a small (6%) increase in BSI for each one‐unit increase in BEI (95% CI 2% – 10%, *p* = 0.002). AWPi10 is also associated with BSI (IRR 3.1, 95% CI 1.20–7.71, *p* = 0.017), with smoking status also associating.

#### 3.5.3. Comparison of YACTA Variables Between Genotypes and Between Those With Pulmonary Functional Impairment

There were no differences between severe (ZZ, FZ, MMalton‐Null and Z‐Null) and nonsevere AATD genotypes (SZ, SS and MZ) in either BEI (BEI, mean 1.15 [1.94] vs 0.67 [0.53], *p* = 0.38), AWPi10 (mean 0.19 [0.08] vs 0.18 [0.08], *p* = 0.73) or PD15 (mean −971.18 [267.34] vs −919.31 [47.58], *p* = 0.487).

BEI was lower in those with COPD compared to those without (mean 0.91 [1.36] vs 1.73 [2.89], *p* = 0.02), though AWPi10 and PD15 did not differ. PD15 was lower in those with KCO < 80% vs ≥ 80% predicted (−965.18 [22.38] vs −926.46 [35.57], *p* < 0.001), though BEI and AWPi10 did not differ.

**FIGURE 7:**
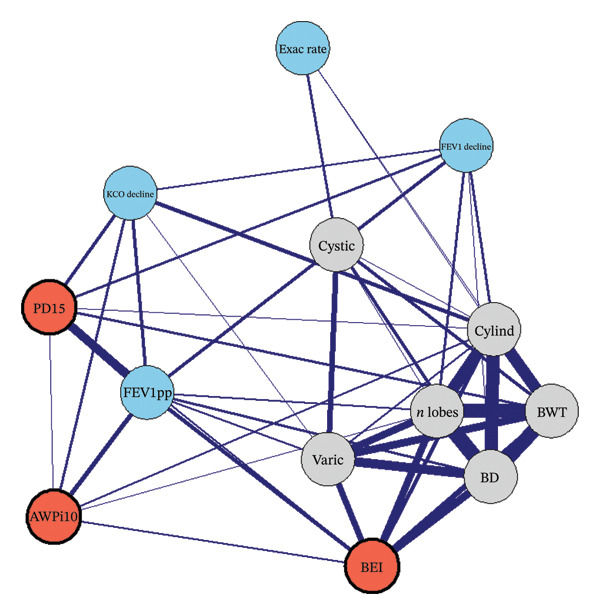
Network diagram demonstrating the relationships between algorithm parameters (red), visual parameters (grey) and clinical outcomes (blue). Lines weighted by strength of correlation. BEI = algorithm airway tapering parameter; PD15 = algorithm emphysema parameter; AWPi10 = algorithm bronchial wall thickness parameter. Exac rate = annualised exacerbation rate; FEV1pp = baseline FEV_1_% predicted; FEV1/KCO decline = annualised FEV_1_/KCO decline, respectively; varic/cylind/cystic = visual identification of varicose/cylindrical/cystic bronchiectasis respectively; BD = visual bronchial dilatation score; BWT = visual bronchial wall thickness score; n lobes = number of lobes with bronchiectasis on visual assessment.

A summary of the associations of the YACTA variables with visual parameters, and with clinical outcomes, is shown in Figure 7.

## 4. Discussion

The aim of this study was to evaluate the feasibility and clinical utility of using YACTA software for the quantitative analysis of bronchiectasis in routine CT scans of patients with AATD. Quantitative analysis of bronchiectasis using YACTA software was both feasible and clinically useful in our cohort of patients who have AATD. Sensitivity to detect disease was adequate, but specificity only reached appropriate thresholds for varicose and cystic morphology. Since these are the most severe types and cystic disease predisposes to decline, this may be useful for risk stratification. Statistically significant relationships between the YACTA BEI and BSI suggest its clinical utility.

### 4.1. Algorithmic CT Bronchiectasis Analysis

Although not all CT scans met criteria for inclusion based on slice thickness, a significant number did, and a good range of bronchiectasis morphology and severity was included. However, as in the visual assessment, only a small minority had cystic or widespread disease, reducing the statistical power of modelling in some cases. A minority of scans were from rare variants: the inclusion of these patients in statistical analysis increases the validity of the data in application to such patients, who are otherwise rarely studied.

#### 4.1.1. Correlation of YACTA Data With Visual Assessment of Bronchiectasis

BEI, the measure of lack of tapering on YACTA, correlated with visual assessment of BD, BWT score, morphology and number of lobes affected. However, caution is required in interpreting these associations, as they were generally weak.

AWPi10 did not correlate with BWT score, which is consistent with Cohen’s κ analysis in our previous work [9], in which there was only a moderate level of agreement between physician and radiologist. BWT may therefore be a difficult parameter for a physician to assess by eye and certainly appears so from available literature [13]. Furthermore, AWPi10 calculates the wall area in comparison to the lumen, but in the case of bronchiectasis, there is both an increased lumen and increased wall area, which can mean the ratio between the two appears normal. AWPi10 has been validated in other studies, and in this study showed some utility in the identification of ever‐smokers.

#### 4.1.2. Thresholds at Which YACTA Data Correlates With Visual Assessment of Bronchiectasis

Secondly, several thresholds were identified at which BEI and AWPi10 were associated with various visual parameters. For many of these, sensitivity and specificity were too low to be practically useful, although a cutoff for BEI of 1.07 seemed to identify BD of 2 × the arterial diameter, 4 or more lobe involvement, and varicose/cystic bronchiectasis, suggesting 1.07 may be the most useful threshold to use in practice. The sensitivity of this value is more important than the specificity, as this will identify a greater proportion of patients, and any false positives could be addressed by visual assessment by a radiologist.

Furthermore, the abilities of BEI to associate with the visual parameters of BD score of ≥ 2, ≥ 4 lobe involvement, and the presence of varicose or cystic bronchiectasis achieved adequate sensitivity and specificity according to published principles for high‐quality clinical testing [34] and at a similar standard to lung cancer screening in the majority of studies in that field [35]. It is known that bronchiectasis involving multiple lobes, or of cystic morphology, is associated with hospitalisation and mortality [11, 12]. With refinement, the thresholds identified here may conceivably lead to efficiencies in radiology reporting by identifying patients who are likely to have these most clinically relevant radiological patterns. Only a small number of our patients had exactly 3 lobes affected, which may have reduced the ability of the models and ROC analysis to identify an optimal threshold for 3 or more lobes, as Chalmers et al. did [12], but studies of larger numbers of CT scans may be able to improve on this. Indeed, published validation data for YACTA shows that certain parameters may have clinical relevance: airway wall thickness and bronchial diameter in the intermediate airways were negatively correlated with FEV_1_pp in adults with cystic fibrosis in one small study [21], and wall thickness has also been negatively correlated with FEV_1_pp in COPD patients [36], although wall thickness is only a nonspecific sign of bronchiectasis and is also present in chronic bronchitis [37].

#### 4.1.3. Correlation of YACTA Data With Clinical Outcomes

Thirdly, the relationship of YACTA parameters with clinical outcomes was assessed. The correlation of BEI with baseline FEV_1_pp was statistically significant but is unlikely to be clinically relevant according to current consensus on minimum clinically important differences for spirometry, where a 5%–10% change is the range of thresholds used for drug development [38], and 6% variability is seen in the majority of individuals [39]. Additionally, the direction of the association is unexpected: patients with bronchiectasis appear to have slightly better baseline FEV_1_pp. Furthermore, BEI was lower in those with COPD. This could be explained by the degree of airways obstruction on referral to tertiary services: those with bronchiectasis, or bronchiectasis‐COPD overlap, are likely to have less severe airways obstruction at referral. Airways obstruction only develops later in the natural history of bronchiectasis, and instead the core symptoms of bronchiectasis, such as exacerbations or phlegm production, are likely to prompt referral in these cases. AWPi10 correlated more strongly with baseline FEV_1_pp and with a negative relationship but did not correlate with lung function decline. However, as a measure of airway thickness, AWPi10 is not specific as a marker of bronchiectasis, and may represent chronic bronchitis in many cases. As a constituent part of COPD, chronic bronchitis will have a direct relationship with baseline FEV_1_pp in these cases.

PD15 was lower in those with gas transfer impairment, which fits with long‐standing knowledge that emphysema impairs gas exchange.

BEI also correlated with BSI score, despite not correlating with exacerbations. This may simply be because of the relationship between BEI and the number of lobes affected, and morphology, both of which feature in the radiology section of the BSI.

### 4.2. Comparison With Similar Algorithms

Although this study was not structured to allow direct comparison with other algorithms, the literature describes several instances of automated algorithms being used to quantify bronchiectasis. Research published by Pieters et al. [40] using the LungQ algorithm has found similar levels of correlation between visual and automated assessment, albeit via a different visual scoring system, but unlike the work presented here, they found BWT correlated with airflow obstruction. Furthermore, their algorithm assessed bronchiectasis only at fixed points of the bronchial tree, rather than the totality as used in our work, and used the broncho‐arterial ratio rather than the tapering assessment in our work. As discussed in our introduction, the use of the broncho‐arterial ratio raises the possibility of false negatives with conditions causing dilated pulmonary arteries, namely pulmonary hypertension, which co‐exists with bronchiectasis in many cases [15].

Work by Cheung et al. [41] using the AirQuant algorithm has been able to assess tapering of the airway, albeit across a more limited level of airway generation than YACTA. As in our case, they did not find a correlation between automatic measures of tapering and clinical outcomes of spirometry or gas transfer but did find an association with mortality. However, theirs was a cohort of patients with idiopathic pulmonary fibrosis (IPF), and AirQuant has been developed for IPF specifically. Such patients can be expected to have a significant burden of traction bronchiectasis, a very different pathophysiology to other bronchiectasis, whereby airways are dilated by the traction generated by fibrosing interstitium.

### 4.3. Strengths and Limitations

Strengths include the level of detail available from YACTA, which is rare among radiological algorithms in being able to create a 3‐dimensional model of the bronchial tree. Automatic algorithms are increasingly used in research of lung diseases, but their main validated utility has so far been the assessment of lung density rather than the airways [42]. Perossi et al. demonstrated the association of algorithm‐derived luminal area and AWPi10 with poorer spirometry, impulse oscillometer measurements and BSI scores [30]. However, they did not use the BEI used here, which requires further validatory studies.

A further strength of the YACTA approach is its objective assessment of BD by analysis of bronchial tapering, which is distinct from other algorithms which have used airway‐artery ratio, as is common in visual assessment [43, 44]. As mentioned in the introduction, assessment of bronchiectasis relative to the accompanying artery is open to bias based on dilatation or constriction of the artery. Use of automated analysis of tapering such as YACTA means these biases can be avoided, and the previously time‐consuming tapering analysis may be performed at scale.

The cohort of patients used here was large but could be further improved in consultation with larger datasets such as the European Alpha‐1 Research Collaboration (EARCO). At present EARCO codes for the presence and morphology of bronchiectasis [7] but does not store anonymised images; storage of such large amounts of data whilst maintaining confidentiality would present logistical challenges, but with technological advances in data storage and security, this may be achievable.

Although all scans used here were of patients reporting a stable, nonexacerbation state, an interesting area of future research could be to assess whether there are unique features of exacerbation detectable by automated analysis, including changes in airway calibre or wall thickness, and whether such changes could associate with exacerbation frequency or severity.

Limitations of all models in this analysis include the correlation coefficients, which were at best only moderate and, in some cases, rather weak. Random variation will account for some of the remaining variability, but other contributing factors may include early childhood exposures [45], occupation and exercise habits, which are not stored reliably in the database.

Variability in CT scanning may have influenced our results. CT scans were acquired over a prolonged period, with imaging protocols and equipment changing over time, and detailed information regarding scanner variability was not consistently available. Although we restricted analysis to scans with slice thickness ≤ 1.5 mm to improve consistency, other factors may have influenced QCT measurements, particularly airway metrics and lung density. This 1.5 mm criteria may also have introduced selection bias by excluding older scans and, therefore potentially patients with more advanced disease or a higher smoking prevalence.

Thresholds derived using Youden’s J method were not validated internally, as all available data were used for model development and may therefore be subject to optimism bias. These thresholds should therefore be considered exploratory and will require validation in independent populations, ideally involving multiple centres.

Additionally, many statistical models were constructed on the same patients, without correction for multiplicity. However, in the context of a rare disease, conventional solutions may increase the risk of missing true relationships. Therefore, statistically significant findings should instead be interpreted cautiously in the context of biological plausibility or consistency with existing literature.

The dataset used in this work consists of selected patients from a centre with specialised expertise in bronchiectasis and AATD. Given that bronchiectasis is often overlooked, automated analysis might be particularly beneficial in centres with less radiological expertise, as it could help in detecting bronchiectasis more effectively.

### 4.4. Potential Applications

The findings of this work suggest that CT analysis of airway tapering in bronchiectasis is sufficiently accurate and could therefore be used to shorten the time required for detailed scan reporting. Although further clinical validation is needed, this approach could potentially increase CT reporting capacity, which may not otherwise keep pace with the growing availability of CT scanning. If successful, this may lead to shorter waiting times for diagnosis. Thresholds here were chosen on the basis of sensitivity rather than specificity, but any false positives could be addressed by a short review by a radiologist, in the manner of recent lung cancer screening programmes [46].

Furthermore, the objective nature of tapering assessment in this algorithm could help standardise the diagnosis of bronchiectasis, which is currently at least partly subjective [13]. This, in turn, may reduce the risk of both over‐ and underdiagnosis, and thereby help avoid inappropriate treatment.

## 5. Conclusions

Algorithmic analysis of bronchiectasis may be a useful tool in AATD to objectively describe airway tapering and wall thickness, since it correlates with visual assessment and BSI score. However, algorithmic bronchiectasis data alone does not relate to clinical outcomes in AATD.

## Funding

This study was supported by the Vertex Pharmaceuticals.

## Conflicts of Interest

Alice M. Turner reports a relationship with CSL Behring, Grifols, Vertex, Takeda, Chiesi, AstraZeneca, GSK, Sanofi and Boehringer Ingelheim that includes: consulting or advisory and funding grants. The other authors declare no conflicts of interest.

## Supporting Information

Additional supporting information can be found online in the Supporting Information section.

## Supporting information


**Supporting Information** Supporting 1 provides detail of the regression models referred to in the results section: “comparison of algorithmic data with clinical outcomes”.

## Data Availability

The data that support the findings of this study are available from the corresponding author upon reasonable request.
