# Supplementary material for: Quantitative Analysis of Bronchiectasis in Alpha‐1 Antitrypsin Deficiency
Source: Can Respir J. 2026 Jun 24;2026:5419612. doi: 10.1155/carj/5419612 (PMC13292108; doi:10.1155/carj/5419612)
Supplement: Supplementary file 1 — Supporting Information Supporting 1 provides detail of the regression models referred to in the results section: “comparison of algorithmic data with clinical outcomes”. [file CARJ-2026-5419612-s001.docx]

Supplement 1

Supplementary Table 1: Linear regression analysis of YACTA parameter association with baseline FEV1 % predicted value. Age and sex are already accounted for in the GLI method for FEV1pp calculation and are therefore omitted.

|  | **Baseline FEV_1_pp** | | |
| --- | --- | --- | --- |
| *Associations* | *Estimates* | *CI* | *p* |
| BEIndexLung | 2.78 | 0.95 – 4.61 | **0.003** |
| AWPi10 | -51.09 | -95.74 – -6.44 | **0.025** |
| PD15 | 0.01 | -0.00 – 0.02 | 0.107 |
| Ever smoker [Yes] | -30.69 | -38.86 – -22.52 | **<0.001** |
| Observations | 133 | | |
| R^2^ / R^2^ adjusted | 0.444 / 0.427 | | |

Supplementary Table 2: Multivariate Poisson regression analysis of algorithm parameters associations with clinical bronchiectasis severity index (BSI). Age is omitted as already included in BSI.

|  | **BSI** | | |
| --- | --- | --- | --- |
| *Associations* | *Incidence Rate Ratios* | *CI* | *p* |
| BEIndexLung | 1.06 | 1.02 – 1.10 | **0.002** |
| AWPi10 | 3.10 | 1.20 – 7.71 | **0.017** |
| PD15 | 1.00 | 1.00 – 1.00 | 0.316 |
| Sex [Male] | 1.13 | 0.97 – 1.33 | 0.119 |
| Ever smoker | 1.42 | 1.17 – 1.73 | **<0.001** |
| Observations | 138 | | |
| R^2^ Nagelkerke | 0.212 | | |
